# Strand invasion by HLTF as a mechanism for template switch in fork rescue

**DOI:** 10.1093/nar/gkt1040

**Published:** 2013-11-05

**Authors:** Peter Burkovics, Marek Sebesta, David Balogh, Lajos Haracska, Lumir Krejci

**Affiliations:** ^1^Institute of Genetics, Biological Research Center, Hungarian Academy of Sciences, HU-6726 Szeged, Hungary, ^2^Department of Biology, Masaryk University, Brno, Czech Republic, ^3^National Centre for Biomolecular Research, Masaryk University, Brno, Czech Republic and ^4^International Clinical Research Center, Center for Biomolecular and Cellular Engineering, St. Anne’s University Hospital Brno, CZ-62500 Brno, Czech Republic

## Abstract

Stalling of replication forks at unrepaired DNA lesions can result in discontinuities opposite the damage in the newly synthesized DNA strand. Translesion synthesis or facilitating the copy from the newly synthesized strand of the sister duplex by template switching can overcome such discontinuities. During template switch, a new primer–template junction has to be formed and two mechanisms, including replication fork reversal and D-loop formation have been suggested. Genetic evidence indicates a major role for yeast Rad5 in template switch and that both Rad5 and its human orthologue, Helicase-like transcription factor (HLTF), a potential tumour suppressor can facilitate replication fork reversal. This study demonstrates the ability of HLTF and Rad5 to form a D-loop without requiring ATP binding and/or hydrolysis. We also show that this strand-pairing activity is independent of RAD51 *in vitro* and is not mechanistically related to that of another member of the SWI/SNF family, RAD54. In addition, the 3′-end of the invading strand in the D-loop can serve as a primer and is extended by DNA polymerase. Our data indicate that HLTF is involved in a RAD51-independent D-loop branch of template switch pathway that can promote repair of gaps formed during replication of damaged DNA.

## INTRODUCTION

Cellular DNA is continuously damaged by numerous endo- and exogenous agents, which, if left unrepaired can cause replication fork stalling and result in gaps and double-strand breaks in the newly synthesized DNA (1). The post-replication repair (PRR) mechanisms have evolved to overcome these discontinuities (2,3). It not only acts in the S-phase, when the majority of DNA has been replicated, but also in the G2 phase (4,5). The PRR mechanism characterized by *RAD6/RAD18*-dependent pathway can bypass the DNA lesions either directly by translesion synthesis polymerases, which can incorporate nucleotides opposite the damaged bases (6–12) or indirectly by template switching (13–15), which facilitates copying from the newly synthesized strand of the sister duplex. The PRR process is also linked with homologous recombination (HR) that could provide an alternative to the *RAD6/RAD18*-dependent pathway means for eliminating discontinuities in newly replicated DNA (16–18).

The HR mechanism is based on the ability of RAD51 protein to form a pre-synaptic filament on single-stranded DNA (ssDNA) with the help of accessory proteins, such as BRCA2, RAD54 and RAD51 paralogues (17,19). This nucleoprotein filament then searches for the homologous region on the sister chromatid, leading to the formation of a D-loop structure. The D-loop is then extended by various DNA polymerases (20–23), followed by either displacement of the extended invading strand and then annealing with the second end [the so-called synthesis-dependent strand-annealing (SDSA) mechanism] (24) or stabilization of the D-loop by second-end capture and formation of a double Holliday junction. Whereas the SDSA pathway leads to generation of gene conversion products, resolution of double Holliday junctions can lead to both gene conversions as well as crossovers (17,25). Although HR constitutes an error-free damage tolerance pathway, the intermediates formed during this process are toxic because they can trigger cell-cycle arrest and cell death (26–28), and HR is thus under very tight control (17,29,30).

In yeasts, *RAD5*-dependent template switching provides alternate mechanisms for the non-recombination branch of PRR (15,31). Rad5, a double-stranded DNA (dsDNA) translocase, can utilize the newly synthesized DNA strand of sister duplex as a template for repair. During this process, a new primer–template junction can be formed by two mechanisms: replication fork regression and template switching initiated by strand invasion (32). Although biochemical studies indicate that Rad5 can regress replication forks by generating four-way Holliday structures, the so-called ‘chicken foot’ intermediates (14), there is no evidence for Rad5 involvement in facilitating strand invasion-mediated template switching. Even though the deletion of *RAD5* gene causes a much higher ultraviolet (UV) sensitivity than point mutations in ATPase domain (33), it suggests additional role for Rad5 protein. In human cells, HLTF, an orthologue of yeast Rad5, possesses ubiquitin ligase and dsDNA translocase activities, as revealed by cell biological and biochemical studies (13,34). HLTF’s functional similarities are evident from its ability to partially complement sensitivities of *rad5* deficient strain (34). HLTF has furthermore been suggested as a potential tumour suppressor gene, as it was found to be silenced in various cancer types (35) and associated with the first stages of carcinogenesis (36). This role is further supported by elevated chromosome breaks and fusion after methyl methanesulfonate (MMS) treatment in HLTF-deficient mouse fibroblast (37).

In this study, we report that HLTF facilitates DNA strand invasion and formation of a D-loop structure in an ATP-independent manner. In the formed D-loop, the 3′-end of the invading strand can be used by a polymerase for its extension. Moreover, the HLTF-dependent D-loop formation and its consequent extension are not dependent on the classical HR enzymes such as RAD51 and RAD54. In contrast to RAD51, HLTF is only partially inhibited by Replication protein A (RPA). Based on these results, we discuss the possibility that HLTF can facilitate strand invasion-dependent mechanisms such as SDSA in addition to fork regression for the template switching.

## MATERIALS AND METHODS

### Plasmids and protein purification

For expressing GST-HLTF and GST-FLAG-HLTF, the HLTF gene was cloned in fusion with glutathione S-transferase (GST) and FLAG tag under the control of the *Saccharomyces cerevisiae* galactose-inducible phosphoglycerate promoter using the Gateway cloning system (Invitrogen) to generate plasmids BIL1000 and BIL1520, respectively. GST-HLTF and FLAG-HLTF were expressed and purified as described previously (34).

Yeast yRad51, yRFC, yPCNA and yDNA polymerase delta were expressed and purified as described previously (21). yRad54 and yRad5 were purified according to the previous publication by Matulova *et al.* (38) and Blastyak *et al.* (14), respectively. Human RAD51 and RAD54 and RPA were purified as described previously (22).

### D-loop assay

The D-loop assay was performed essentially as described previously (21). A radioactively labelled 90-mer (3 μM nucleotides, D1 oligonucleotide, 5′- AAA TCA ATC TAA AGT ATA TAT GAG TAA ACT TGG TCT GAC AGT TAC CAA TGC TTA ATC AGT GAG GCA CCT ATC TCA GCG ATC TGT CTA TTT -3) complementary to positions 1932–2022 of the pBluescript replicative form I DNA was incubated for 3 min at 37°C with HLTF (200 nM) in 10 μl of buffer R [35 mM Tris–Cl, pH 7.4, 2 mM ATP, 2.5 mM MgCl_2_, 50 mM KCl, 1 mM DTT and an adenosine triphosphate (ATP)-regenerating system consisting of 20 mM creatine phosphate and 20 μg/ml creatine kinase]. In case of Rad5 the incubation was carried out at 30°C. Alternatively and as indicated, a D2 oligonucleotide (5′- ATC AGC TCA CTC AAA GGC GGT AAT ACG GTT ATC CAC AGA ATC AGG GGA TAA CGC AGG AAA GAA CAT GTG AGC AAA AGG CCA GCA AAA GGC -3′) complementary to positions 1191–1180 or a D0 oligonucleotide (5′- ATC CGC TAG CGA CCA TGG GCA GCA GCC ATC ATC ATC ATC ATC ACA GCA GCG GCG AAA ACC TGT ATT TTC AAG GCA TCG ATG ATT ACA AGG ATG AC -3′) having no sequence similarity to the pBSK plasmid DNA sequence was used instead of the D1 oligonucleotide. Gapped DNA substrate was generated using radioactively labelled D1 oligonucleotide and C1 (5′-CTC ATA TAT ACT TTA GAT TGA TTT-3′) and C2 (5′-AAA TAG ACA GAT CGC TGA GAT AGG-3′) oligonucleotides. The reactions were initiated by adding 2 μl of pBluescript replicative form I (50 μM base pairs) followed by incubation for 10 min at 37°C. The reactions were stopped by addition of proteinase K (0.5 mg/ml) and sodium dodecyl sulphate (SDS; 0.5%) after 20 min incubation and loaded onto 0.8% (w/v) agarose gel. After electrophoresis, the gel was dried on DE81 paper and exposed on a phosphorimager screen. Quantification of the results was done using a Fuji FLA 9000 imager (Fuji), followed by analysis with MultiGauge software (Fuji).

### DNA extension

The reactions were performed as described previously (21). Briefly, the D-loop reaction (10 μl), RPA (660 nM), proliferating cell nuclear antigen (PCNA) (6.66 nM), yeast Replication factor C (yRFC) (10 nM) and yPol δ (16 nM) were mixed in buffer O (20 mM Tris–Cl, pH 7.5, 5 mM DTT, 0.1 mM EDTA, 150 mM KCl, 40 μg/ml BSA, 8 mM MgCl_2_, 5% glycerol, 0.5 mM ATP and 75 μM each of dGTP and dCTP), followed by 5 min incubation at 30°C. DNA synthesis was initiated by adding start buffer (75 μM dTTP and 75 μM dATP in buffer O) with 30 μl final reaction volume. When using an unlabelled D1 oligonucleotide, 75 μM dTTP and 0.375 μCi [α-^32^P]dATP in buffer O was used as a start buffer. The reactions were stopped by addition of proteinase K (0.5 mg/ml) and SDS (0.5%) after 10 min at 30°C and loaded onto 0.8% (w/v) agarose gel. After electrophoresis, the gel was dried on DE81 paper and exposed on a phosphorimager screen. Quantification of the results was done using a Fuji FLA 9000 imager (Fuji), followed by analysis with MultiGauge software (Fuji).

### Topoisomerase I-linked assay

Relaxed DNA was prepared by incubating 5 μg of ΦX 174 replicative form DNA with 5 U of *Escherichia coli* topoisomerase I (NEB) in 100 μl of NEB4 buffer for 2 h at 37°C. DNA was then purified by phenol extraction and ethanol precipitation and dissolved in TE buffer (10 mM Tris-Cl, 1 mM EDTA, pH 7.5) to a concentration of 100 ng/μl. The indicated amounts of HLTF protein were incubated with 100 ng of relaxed DNA (27.3 μM nucleotides) in 10 μl R buffer (35 mM Tris–Cl, pH 7.4, 2 mM ATP, 5 mM MgCl_2_, 50 mM KCl, 1 mM DTT, 50 mM NaCl, 1 mM ATP) for 10 min at 37°C, followed by the addition of 1 U of calf thymus topoisomerase I. The incubation was continued for 10 min at 37°C. The reactions were stopped by addition of proteinase K (0.5 mg/ml) and SDS (0.5%) for 10 min at 37°C and loaded onto 0.8% (w/v) agarose gel. After electrophoresis, the gel was stained by ethidium bromide and visualized with Typhoon TRIO imager.

### GST pull-down experiment

Purified GST or GST-HLTF proteins (3 μg) were incubated with Glutathione-Sepharose (GTH) beads (GE Healthcare) for 4 h at 4°C with FLAG-HLTF (1 μg) in buffer E (40 mM Tris–HCl, pH 7.5, 100 mM NaCl, 0.1 mM DTT, 10% glycerol, 0.01% NP40). Beads were washed three times with buffer E and bound proteins were eluted with buffer E containing 20 mM reduced glutathione. Elution fractions were analysed by 10% SDS–PAGE (polyacrylamide gel electrophoresis) followed by coomassie blue staining or immunoblotting using anti-FLAG antibody.

## RESULTS

### HLTF and Rad5 are able to facilitate D-loop formation

**Figure 1.**
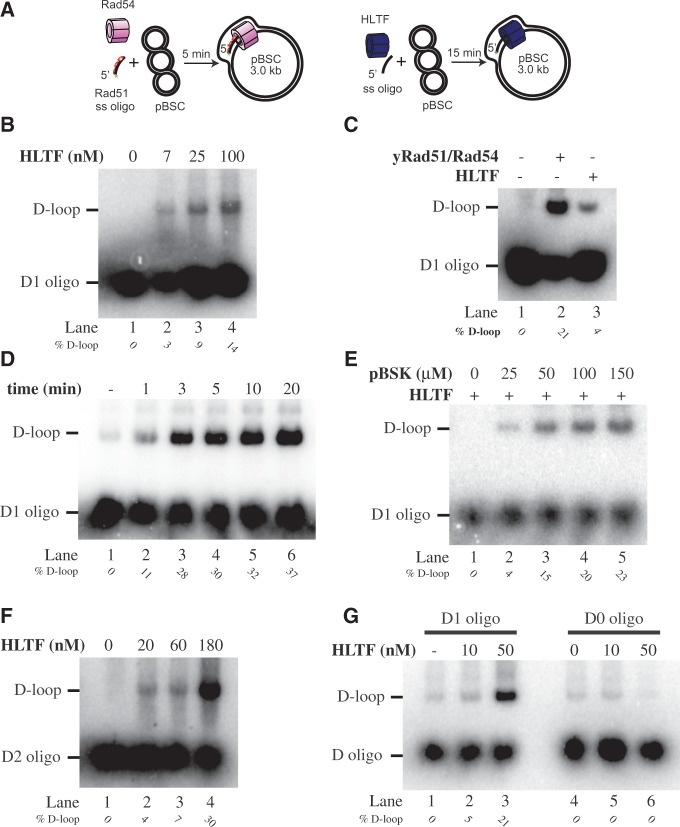
HLTF catalyses D-loop formation. (**A**) Schematic representation of RAD51- and HLTF-mediated D-loop reactions. (**B**) Concentration-dependent formation of the D-loop by HLTF. Increasing amounts of HLTF were incubated with the radioactively labelled D1 oligonucleotide. After addition of the pBSK plasmid DNA, the reactions were incubated for 20 min at 37°C and analysed. (**C**) D-loops generated by human HLTF (100 nM) and yRad51/yRad54 (1 µM/150 nM, respectively) are indistinguishable. The yRad51 or for HLTF were pre-incubated with the D1 oligonucleotide at 37°C for 5 min, after which yRad54 or buffer was added. After 3 min incubation at 23°C, the pBSK plasmid DNA was added and incubated for 5 min at 23°C. (**D**) Kinetics of HLTF-mediated D-loop activity. HLTF (100 nM) and D1 oligonucleotide (3 µM nucleotides) were incubated at 37°C with aliquots withdrawn at the indicated time. (**E**) D-loop formation by HLTF is dependent on the concentration of homology-containing plasmid DNA. The HLTF was incubated with the D1 oligonucleotide followed by addition of increasing concentrations of pBSK plasmid DNA (0–150 µM base pairs). (**F**) HLTF-mediated D-loop formation is not sequence specific. Increasing amounts of HLTF (10 and 50 nM) were incubated with D2 oligonucleotide, another pBSK homology-containing sequence. (**G**) D-loops formation by HLTF requires sequence homology. The D-loop reactions used oligonucleotide containing (D1 oligo) or lacking (D0 oligo) sequence homology to pBSK plasmid. All reactions were stopped by addition of SDS/proteinase K and reaction products were resolved on 0.8% native agarose gel. The positions of free oligonucleotide and D-loops are indicated together with the quantification of D-loops (as percentages of formed D-loop versus free oligonucleotide).

To examine whether HLTF is able to facilitate template switching not only by fork regression but also by strand invasion, we tested the ability of HLTF to form D-loop (Figure 1A). Incubation of HLTF with the D1 oligonucleotide and the donor plasmid DNA containing the region of homology resulted in the concentration-dependent appearance of a slowly migrating band (Figure 1B). We also compared the HLTF activity with the reaction catalysed by yeast yRad51 and yRad54 proteins, known to form a D-loop structure (Figure 1C). We found that the mobility of the structure generated by yRad51/yRad54 and that by HLTF is indistinguishable. The dependence of the reaction on the incubation time (Figure 1D) and the amount of the supercoiled plasmid (Figure 1E) provided further support that the slowly migrating band is specific to the HLTF activity.

Next, the sequence specificity of the reaction was analysed using a D2 oligonucleotide complementary to a different region of the supercoiled plasmid DNA. As shown in Figure 1F, the D2 was as efficient as the D1 oligonucleotide in the reaction, confirming that HLTF D-loop activity is not dependent on a specific target sequence. Similarly, the control reaction using a non-complementary D0 oligonucleotide (Figure 1G) or non-complementary supercoiled plasmid DNA (Supplementary Figure S1A) resulted in no D-loop formation, thus indicating the ability of HLTF to facilitate strand invasion only into homology-containing DNA.

As Rad5 is yeast HLTF homologue, we next asked whether it is also capable of D-loop formation. As shown in Supplementary Figure S1B, Rad5 was able to generate the D-loop product in concentration-dependent manner, indicating conservation of this activity between yeast and human.

### Extension of the D-loop formed by HLTF

**Figure 2.**
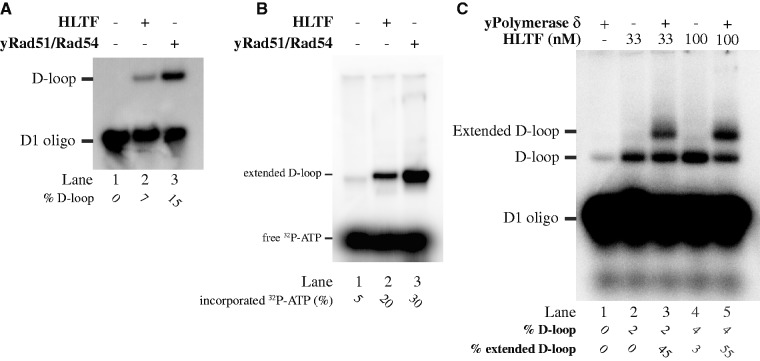
Extension of the HLTF-mediated D-loops. (**A**) Comparison of level of HLTF- or yRad51/yRad54-mediated D-loop formation used for extension reaction in (**B**). A radioactively labelled (panels A and C) or unlabelled (panel B) D1 oligonucleotide was used for the D-loop assay. (B) D-loop generated with unlabelled D1 oligonucleotide was extended by yeast DNA polymerase δ. Extension of the D-loop is monitored by incorporation of ^32^P labelled dATP. (**C**) Radioactively labelled D-loop was used for the D-loop reaction followed by the extension with DNA polymerase δ. Reactions were stopped by addition of SDS/proteinase K and reaction products were resolved on 0.8% native agarose gel. The positions of the free oligonucleotide, D-loop and extended D-loop are indicated together with the quantification of D-loop (as a percentage of D-loop formed versus free oligonucleotide) and their extension products (as a percentage of D-loop extended versus not extended).

**Figure 3.**
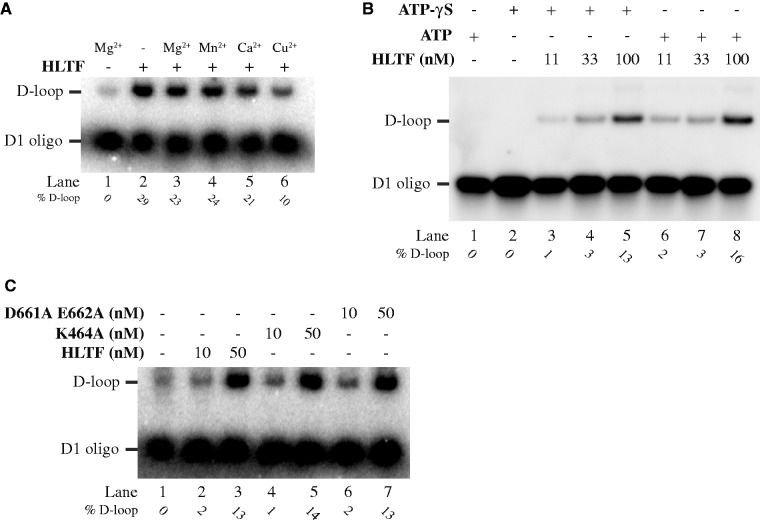
ATPase activity of HLTF is not required for D-loop formation. (**A**) Dependence of D-loop reaction on the presence of metal ions. Reactions containing no protein sample (lane 1) or no metal ion in the reaction buffer (lane 2) constitute control experiments. The remaining reactions were carried out in the presence of 0.5 mM MgCl_2_ (lane 3), 0.5 mM MnCl_2_ (lane 4), 0.5 mM CaCl_2_ (lane 5) or 0.5 mM CuSO_4_ (lane 6), respectively. (**B**) ATP hydrolysis is not required for D-loop formation. The D-loop assay was carried out in the presence of ATP (lanes 3–5) or ATP-γS (lanes 6–8). (**C**) Neither magnesium nor ATP binding is necessary for the HLTF-mediated D-loop activity. The reactions were carried out using 10 and 50 nM of each: wild-type, K464A (Walker A; ATP binding) or D661A E662A (Walker B; magnesium binding) HLTF mutants. All reactions were stopped by addition of SDS/proteinase K and reaction products resolved on 0.8% native agarose gel. The position of the free oligonucleotide and D-loop are indicated together with the quantification of D-loop (as a percentage of D-loop formed versus free oligonucleotide).

Next, we wanted to test whether the 3′-end of the invading strand in the DNA structure formed by HLTF can serve as a primer for a DNA polymerase. We therefore took advantage of our previously described D-loop extension assay using yeast DNA replication machinery, consisting of yPCNA, yRPA, yRFC and yDNA polymerase δ and radioactively labelled dATP (21). D-loops formed either by yRad51/yRad54 or HLTF proteins, respectively, (Figure 2A) were compared in these reactions. As shown in Figure 3B, both HLTF- and yRad51/yRad54-mediated D-loops were extendable and the degree of extension correlated with the amount of the D-loop initially generated (compare Figure 2A and B). Alternatively, we used a radioactively labelled D1 oligonucleotide for D-loop formation and subsequent extension and demonstrate the extension of the HLTF-mediated D-loops by the replication complex (Figure 2C). In summary, these experiments demonstrate that the D-loop formed by HLTF is indistinguishable from that formed by yRad51/yRad54 and is suitable for the downstream DNA extension steps of repair reaction.

### ATPase activity of HLTF is not required for D-loop formation

The Rad51- or RecA-mediated D-loop reaction is an ATP-dependent process (39); therefore, we also tested whether the ATPase activity of HLTF is essential for D-loop formation. Several findings indicated that ATP is not required for HLTF-mediated D-loop activity. First, substitution of Mg^2+^, a cation that coordinates ATP hydrolysis, by Mn^2+^ and Ca^2+^ did not affect the HLTF D-loop activity (Figure 3A). Second, the use of a non-hydrolysable ATP analogue (ATP-γS) also did not influence the reaction (Figure 3B), thus indicating that HLTF does not require ATP hydrolysis for D-loop formation. Finally, the Walker A (K464A) and Walker B (D661A E662A) mutants of HLTF formed D-loops to the same extent as did the wild-type protein (Figure 3C). Taken together, all the experiments demonstrate the ability of HLTF to form D-loops even without binding and/or hydrolysis of ATP.

### Effect of RAD51, RAD54 and RPA on HLTF-promoted D-loop formation

**Figure 4.**
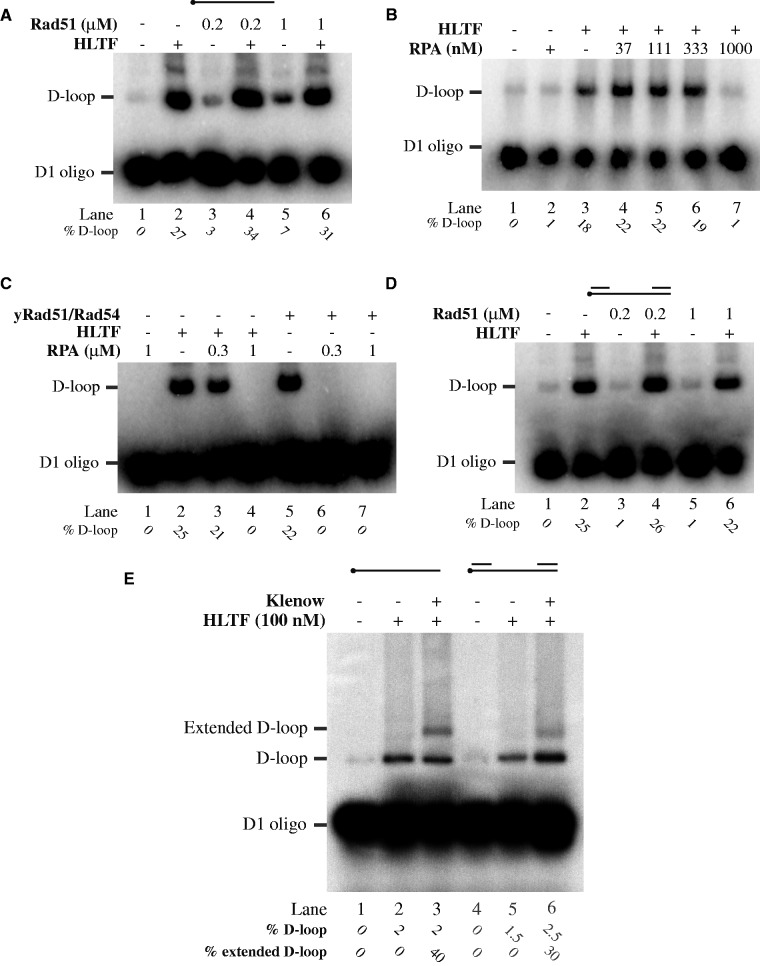
Comparison of HLTF- and RAD51-dependent mechanisms of D-loop formation. (**A**) HLTF and RAD54 have different roles in the D-loop formation. Increasing amounts of RAD51 (0.2 and 1 μM) were used in the presence or absence of HLTF (100 nM), as indicated. (**B**) HLTF D-loop activity is inhibited by RPA. Increasing amounts of RPA were pre-incubated with the D1 oligonucleotide before addition of HLTF (100 nM). (**C**) Comparison of the inhibitory effect of RPA on RAD51- and HLTF-mediated D-loop formation. Saturated and oversaturated amounts of RPA were incubated in the presence or absence of HLTF (100 nM) and yRad51 (1 μM)/yRad54 (150 nM). (**D**) Only HLTF forms the D-loop on gapped DNA substrate. Increasing amounts of RAD51 were incubated in the presence or absence of HLTF (100 nM) on gapped DNA substrate. Reactions were stopped by addition of SDS/proteinase K and resolved on 0.8% native agarose gel. Percentages of D-loop formed are indicated at the bottom of the gels. (**E**) Extension of gaped DNA-mediated D-loop. Radioactively labelled D1 oligo (5 nM) was used for the D-loop reaction followed by the extension with Klenow polymerase (5 nM). Reactions were stopped by addition of SDS/proteinase K and reaction products were resolved on 0.8% native agarose gel. Positions of free oligonucletide, D-loop and extended D-loop are indicated together with the quantification of D-loop (as a percentage of formed D-loops versus free oligonucleotide) and their extension products (as a percentage of extended D-loops versus not extended).

It was reported that RAD54 can significantly enhance RAD51-mediated D-loop formation (40,41). As both HLTF and RAD54 belong to the SWI/SNF family of DNA translocases and share many biochemical properties, we were interested to know if HLTF can facilitate D-loop formation by RAD51. To this end, we compared HLTF-dependent D-loop formation using naked and RAD51-coated D1 oligonucleotide. As shown in Figure 4A, the same amounts of D-loop were generated regardless of the presence or absence of RAD51. Thus, HLTF was not able to substitute RAD54 and stimulate RAD51-dependent D-loop activity. This indicates that HLTF could play a role in RAD51-independent D-loop formation pathway. In addition, the difference between HLTF and RAD54 is further demonstrated by the inability of RAD54 to form the D-loop alone (Supplementary Figure S2B).

Long ssDNA tracks are often coated by RPA *in vivo*, which results in inhibition of the RAD51-dependent D-loop formation and requires action of recombination mediators to replace RPA by RAD51 on ssDNA (17). To gain insight as to whether RPA can inhibit also HLTF-mediated D-loop formation, D1 oligonucleotide was pre-incubated with increasing concentration of RPA before the addition of HLTF. While a saturating concentration of RPA on ssDNA, as confirmed by a gel shift assay (Supplementary Figure S2C), completely inhibited RAD51-dependent D-loop formation, we did not observe inhibition of HLTF activity (Figure 4B). The inhibition of HLTF was noted only at 3-fold higher RPA concentration (Figure 4C), indicating a possibly different mode of action. Next, we asked whether the ATP-dependent protein remodelling activity of HLTF could help suppress the RPA inhibition. However, the ATPase-deficient HLTF mutant was inhibited similarly as wild-type HLTF at oversaturated concentration of RPA (Supplementary Figure S2D), suggesting that this activity is dispensable for the D-loop activity.

To explore this possibility further, RAD51 and HLTF D-loop activities were compared using a gapped DNA, mimicking DNA substrate formed opposite DNA lesions during replication. Strikingly, and in contrast to RAD51 protein (Figure 4D), the HLTF was fully able to efficiently utilize gapped DNA for D-loop formation. Furthermore, the D-loops generated by this substrate were also extendable by a DNA polymerase (Figure 4E), indicating that they are suitable for the DNA repair synthesis step. These findings demonstrate possible differences between RAD51 and HLTF in their abilities to form the D-loop, thus suggesting their roles in alternative pathways.

### Mechanism of D-loop formation by HLTF

**Figure 5.**
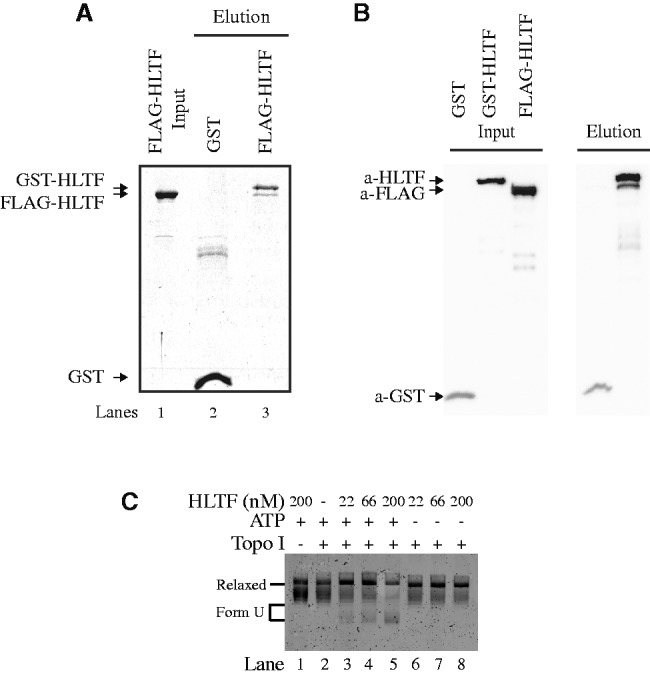
Mechanism of D-loop formation by HLTF. (**A**) Self-association of HLTF. GST pull-down experiment was carried out using FLAG-HLTF and GST-HLTF or GST followed by immobilization on GTH beads. The beads were washed, bound proteins were eluted and reactions were analysed on 10% SDS–PAGE gels. (**B**) Western blot analyses of the GST pull-down experiment. We sequentially developed the filter by anti-FLAG, anti-GST and finally anti-HLTF antibodies as indicated on the bottom of the gels together with the bands corresponding to individual proteins. (**C**) HLTF mediates change in DNA conformation in ATP-dependent manner. Increasing amounts of HLTF (22, 66 and 200 nM) were incubated with topologically relaxed DNA and topoisomerase I with (lanes 2–5) or without (lanes 6–8) ATP as indicated. Lane 1 represents control reaction in the absence of topoisomerase I. All reactions were stopped by addition of SDS/proteinase K and reaction products resolved on 0.8% native agarose gel. Position of Relaxed and Form U DNA is indicated.

In order to shed light on the mechanism of D-loop formation, we generated and tested several point and deletion mutants of HLTF. Unfortunately, we were not able to isolate a separation-of-function mutant as the generated truncations also affected other HLTF activities in addition to D-loop formation (Supplementary Figure S3A). As HLTF-mediated D-loop activity is ATP independent, we hypothesized that protein oligomerization (dimerization) might be important in D-loop formation by bridging the invading and donor strands. To test this hypothesis, a pull-down experiment using FLAG- and GST-tagged versions of HLTF protein was conducted. Mixing both proteins with GTH beads resulted in retention of FLAG-HLTF on the beads, indicating self-association of HLTF (Figure 5). Furthermore, DNA binding does not influence this interaction as the addition of plasmid DNA had no effect on the oligomerization (data not shown). In conclusion, HLTF self-association might contribute to D-loop activity.

Another possible mechanism, by which HLTF may facilitate D-loop formation, is the change of DNA topology. By this mean, HLTF may generate topological stress that may facilitate the invasion of ssDNA into the donor dsDNA. Therefore, we tested whether HLTF can alter the topology of the supercoiled plasmid DNA. As shown in the Figure 5C, HLTF indeed induced changes in DNA conformation; however, this activity was fully dependent on ATP. Even-though we observed that HLTF forms D-loop structures independently of ATP *in vitro* (Figure 3), we cannot exclude the possibility that *in vivo*, in the context of chromatinized DNA, both the ATP-dependent and the ATP-independent mechanism have a functional relevance.

## DISCUSSION

Stalling or collapse of the replication fork at DNA lesions leads to gaps being left behind the replicated DNA and can persist until the G2/M phase of the cell cycle (1). Two possible mechanisms for repair of the gapped DNA by template switching have been proposed (32). While the first generates a new template–primer junction by reversal of the replication fork, the second is a result of a strand invasion leading to the D-loop structure (32). Yeast Rad5 and its human orthologue HLTF have been shown to play a major role in providing the continuity of the newly replicated DNA by fork reversal activity (13–15) (Supplementary Figure S3B). Although it is well known that human RAD51 protein can mediate strand invasion and D-loop activity, this study tested such activity for HLTF protein also.

We found that incubation of HLTF with an oligonucleotide and a supercoiled donor plasmid containing homologous DNA resulted in the appearance of a slowly migrating band, similar to the reaction with RAD51 protein. Formation of this DNA structure was proportional to time and amount of HLTF protein, and it required homology between the oligonucleotide and the donor plasmid. Furthermore, to support the view that HLTF-mediated D-loop may constitute a substrate for downstream repair synthesis, we showed that the yeast polymerase δ in the presence of its accessory proteins PCNA and RFC efficiently extends the 3′-end of the invading primer.

In addition, the D-loop activities of RAD51 and HLTF proteins were compared and the effect of ATP and Mg^2+^ was tested. In contrast to RAD51, neither Mg^2+^ nor ATP was required for D-loop formation by HLTF. This difference is further supported by the proficiency in D-loop formation by HLTF mutants that are not expected to bind Mg^2+^ (HLTF D661A E662A) or ATP (HLTF K464A). The ATP-independent role of HLTF is in agreement with study of *RAD5* othologue as its deletion causes a much higher UV sensitivity than point mutations in ATPase domain (33). Indeed, we also observed that Rad5 is capable of D-loop formation and this activity does not require ATP (Supplementary Figure S1B), indicating evolutionary conservation of this mechanism. However, we cannot rule out that *in vivo* the ATPase activity contributes to efficient D-loop formation by removing other DNA-binding factors or inducing topological changes in the DNA.

A possible explanation for different reaction mechanisms is the role of ATP in conformational change that stabilizes Rad51 filament on ssDNA (42), whereas the DNA binding is ATP independent in the case of HLTF (data not shown). The difference between HLTF and RAD51 is further evident from the ability of HLTF, unlike RAD51, to utilize gapped DNA in the D-loop reaction (Figure 4D). As gapped DNA represents discontinuities left opposite DNA lesion during replication, it may closely resemble substrates for the template switching mechanism. Furthermore, despite its similarities to RAD54, another member of the SWI/SNF family of translocases, HLTF cannot stimulate RAD51-dependent D-loop formation. The difference is also apparent from the inability of RAD54 to catalyse the D-loop reaction without Rad51 protein.

In addition, it was observed that RPA bound to the invading DNA fully inhibits Rad51-mediated reactions. That was in contrast to HLTF, which was not inhibited at saturating RPA concentration. We thus speculate that whereas RAD51-dependent reaction requires mediator proteins, HLTF may not be dependent on other accessory factors. The study also shows that the presence of RAD51 does not negatively influence HLTF activity, indicating that RAD51 filament may not present an obstacle for HLTF. We conclude that HLTF facilitates D-loop formation independently of RAD51, thus suggesting HLTF-dependence.

The findings collectively suggest distinct mechanisms for RAD51- and HLTF-catalysed D-loop formation and indicate that HLTF, in addition to fork reversal, could also indicate an alternative pathway for template switching independent of the players of the canonical HR pathway (Supplementary Figure S4). In this model, HLTF is able to repair gaps left behind the replication fork opposite the lesion while the sister strand serves as the donor available for template switch, accordingly to the ability of HLTF to utilize such substrate for D-loop reaction. In addition, HLTF’s ability to oligomerize could allow tethering of the invading and the donor DNA strands. This interaction may result in a topological constraint that leads to a partial opening of the strands of the newly synthesized DNA and promotes the homology search followed by stabilization of the D-loop structure. This mechanism may resemble the one described for TRF2- and Taz1-dependent T-loop (a telomeric D-loop) formation (43,44), with the difference that HLTF joins invading and donor DNA from two different molecules. The extension of the invading strand of the D-loop structure by conventional replication machinery followed by stand displacement and ligation then restores the continuity of DNA.

It has been shown that HLTF suppresses mutagenesis upon treatment of cells with DNA damaging agents like MMS or UV (45) and mice lacking HLTF accumulate broken chromosomes upon MMS treatment (37). It was proposed that this suppression is mediated by PCNA ubiquitilation, which in turn recruits Pol η, thereby assuring error-free bypass of UV lesions (45). However, it is possible that subset of these events may involve template switching. In this scenario, HLTF could promote the error-free bypass of the lesions by promoting D-loop formation. On the other hand, mice cells lacking HLTF are as resistant as wild-type cells to a variety of DNA damaging agents, suggesting a functional overlap with either other functional homologue of Rad5, SHPRH or other repair pathways (46,47). Clearly, more experiments will be required to understand the role of HLTF in genome stability as well as cooperativity and regulation of parallel repair pathways.

In summary, this study has described a D-loop activity for HLTF protein, suggesting an alternate mechanism for HLTF in facilitating template switching that is in addition to its previously described fork reversal activity. HLTF and other members of the SWI/SNF family have been shown to be inactivated in several cancer types (34,48–50). We propose that HLTF might restore the continuity of the gapped DNA by a RAD51-independent mechanism and this may help to explain its possible tumour-suppressor properties.

## SUPPLEMENTARY DATA

Supplementary Data are available at NAR Online.

## FUNDING

Czech Science Foundation [GACR 13-26629S and 207/12/2323]; co-financed from European Social Fund and the state budget of the Czech Republic [CZ.1.07/2.3.00/20.0011]; European Regional Development Fund (Project FNUSA-ICRC) [No. CZ.1.05/1.1.00/02.0123]; Hungarian Science Foundation [OTKA 101225, GOP-1.1.1-11.2012-0384, GOP-1.1.1-11-2011-0026 and GOP-1.1.1-11-2012-0030]. Funding for open access charge: Grant number [CZ.1.07/2.3.00/20.0011] co-financed from European Social Fund and the state budget of the Czech Republic.

*Conflict of interest statement.* None declared.

## Supplementary Material

Supplementary Data
