# Supplementary material for: Strand invasion by HLTF as a mechanism for template switch in fork rescue
Source: Nucleic Acids Res. 2013 Nov 5;42(3):1711–20. doi: 10.1093/nar/gkt1040 (PMC3919600; doi:10.1093/nar/gkt1040)
Supplement: Supplementary Data [file supp_42_3_1711__index.html]

Strand invasion by HLTF as a mechanism for template switch in fork rescue — Strand invasion by HLTF as a mechanism for template switch in fork rescue — Supplementary Data 

# Strand invasion by HLTF as a mechanism for template switch in fork rescue

## Supplementary Data

files

**Files in this Data Supplement:**

- Supplementary Data - pdf file
