# Supplementary material for: Strand invasion by HLTF as a mechanism for template switch in fork rescue
Source: Nucleic Acids Res. 2013 Nov 5;42(3):1711–20. doi: 10.1093/nar/gkt1040 (PMC3919600; doi:10.1093/nar/gkt1040)
Supplement: Supplementary Data [file supp_gkt1040_nar-01616-d-2013-File007.pdf]

SUPPLEMENTARY MATERIAL

Supplementary Figure 1: HLTF requires sequence homology to form D-loop. **A)** HLTF (100 nM) was incubated with D1 oligonucleotide (5 nM) before addition of pBSK or pE3C plasmids containing or missing sequence homology, respectively. **B)** Concentration-dependent D-loop formation by Rad5, yeast homolog of HLTF. Increasing amounts of yRad5 (40 and 120 nM) were incubated with the radioactively labelled D1 oligonucleotide. After addition of the pBSK plasmid DNA, the reactions were incubated for 20 min at 30°C and analysed. All reactions were stopped by addition of SDS/proteinase K and reaction products were analysed on 0.8% native agarose gel. Position of free D1 oligonucleotide and D-loop is indicated.

Supplementary Figure 2: **A)** Comparison of D-loop formation by HLTF and RAD54 (another member of SWI/SNF family) proteins. Increasing amounts of RAD54 or HLTF were used in the D-loop assay. **B)** DNA binding by RAD51. To monitor the DNA binding activity D1 oligonucleotide (5 nM) was incubated with RAD51 (1 μM). Reactions were resolved on 10% native polyacrylamide gel. **C)** RPA binding to ssDNA. Increasing amounts (37, 111, 333, or 1000 nM) of RPA were incubated with D1 oligonucleotide (5 nM) and reactions were resolved on 10% native polyacrylamide gel. **D)** Effect of RPA on D-loop activity of HLTF ATPase mutant. Increasing amounts of RPA (111, 333 and 1000 nM) were pre-incubated with the D1 oligonucleotide before addition of ATPase deficient HLTF mutant (D661AE662A, 100 nM). All reactions were stopped by addition of SDS/proteinase K and reaction products resolved on 0.8% native agarose gel. Position of free D1 oligonucleotide and D-loop is indicated

Supplementary Figure 3: **A)** Schematics of various HLTF truncations and their D-loop activities. Symbol DEF indicates that these mutants are defective not only the D-loop forming but

also ubiquitin-ligase activities. **B)** Summary of the HLTF domains and their corresponding functions. Symbol: ND, **not detected.**

Supplementary Figure 4: Model of the role of HLTF. Stalled or collapsed replication forks result in increased number of gapped DNA or double strand break (DSB) behind the replication fork. DSB repair can either be repaired by RAD51-dependent D-loop formation. Alternatively, it has been demonstrated that HLTF has a fork regression activity and mediate template switch mechanism via fork regression. Furthermore, fork independent template switching mechanism, acting after the fork collapse was also proposed for repair of gapped DNA substrate. Our result suggested that HLTF could mediate this gap repair. In this mechanism, HLTF could bind both strands of the newly replicated DNA and via its self-association could bring dsDNA strands into close proximity and promote strand invasion. This could be further facilitated by topological constrains in the DNA. Switching of the template allows extension of the 3' end by DNA polymerases. The extended D-loop is then displaced and residual 3' flap structure processed by nucleases, followed by ligation of the ends. While HLTF in contrast to RAD51 is able to mediate D-loop formation of gapped DNA, possible crosstalk or overlap of RAD51- and HLTF-dependent mechanism cannot also be excluded.

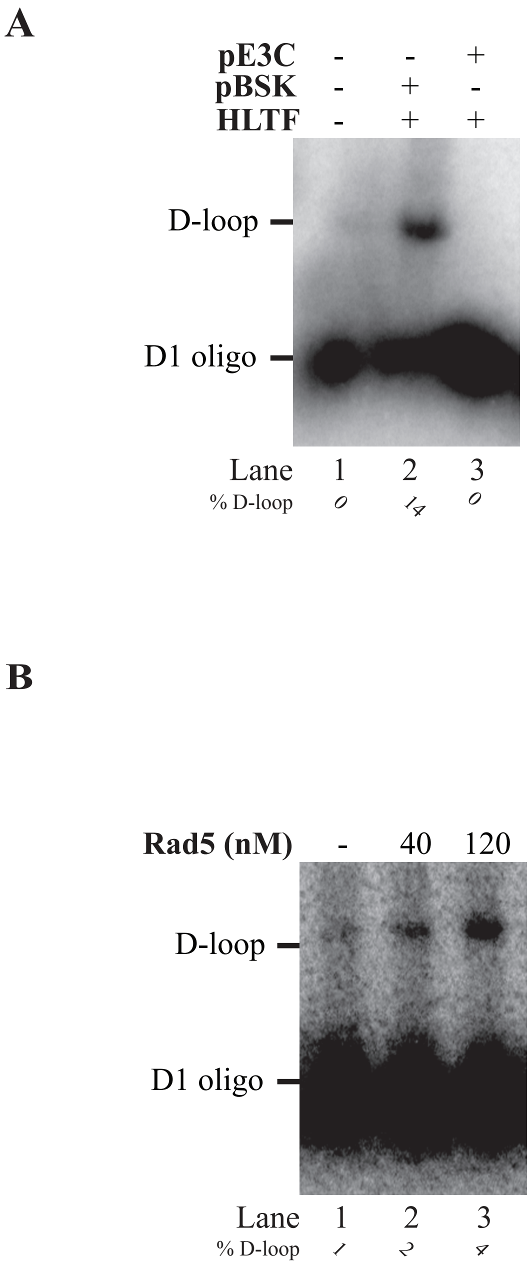

Supplementary Figure 1

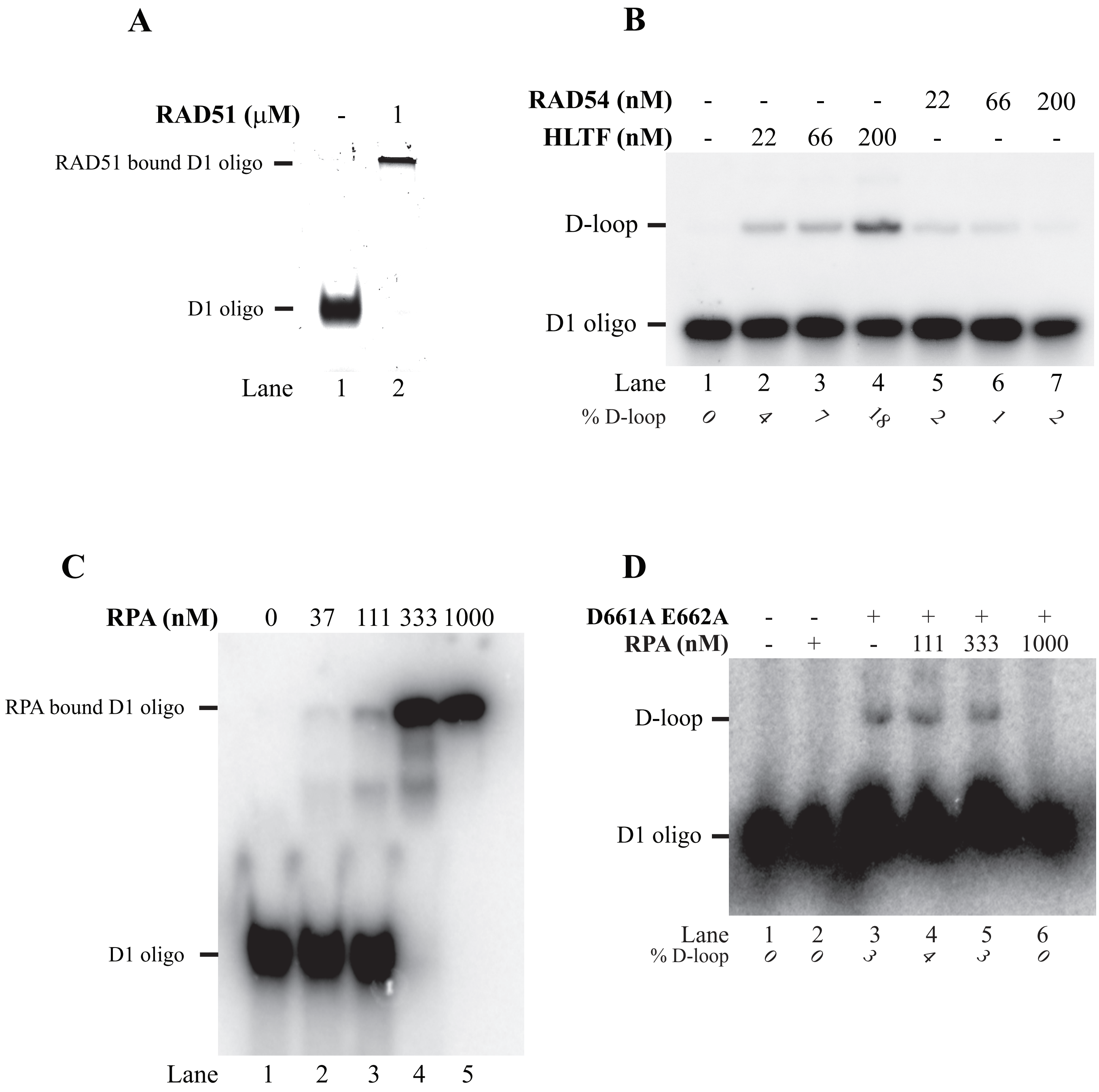

Supplementary Figure 2

A

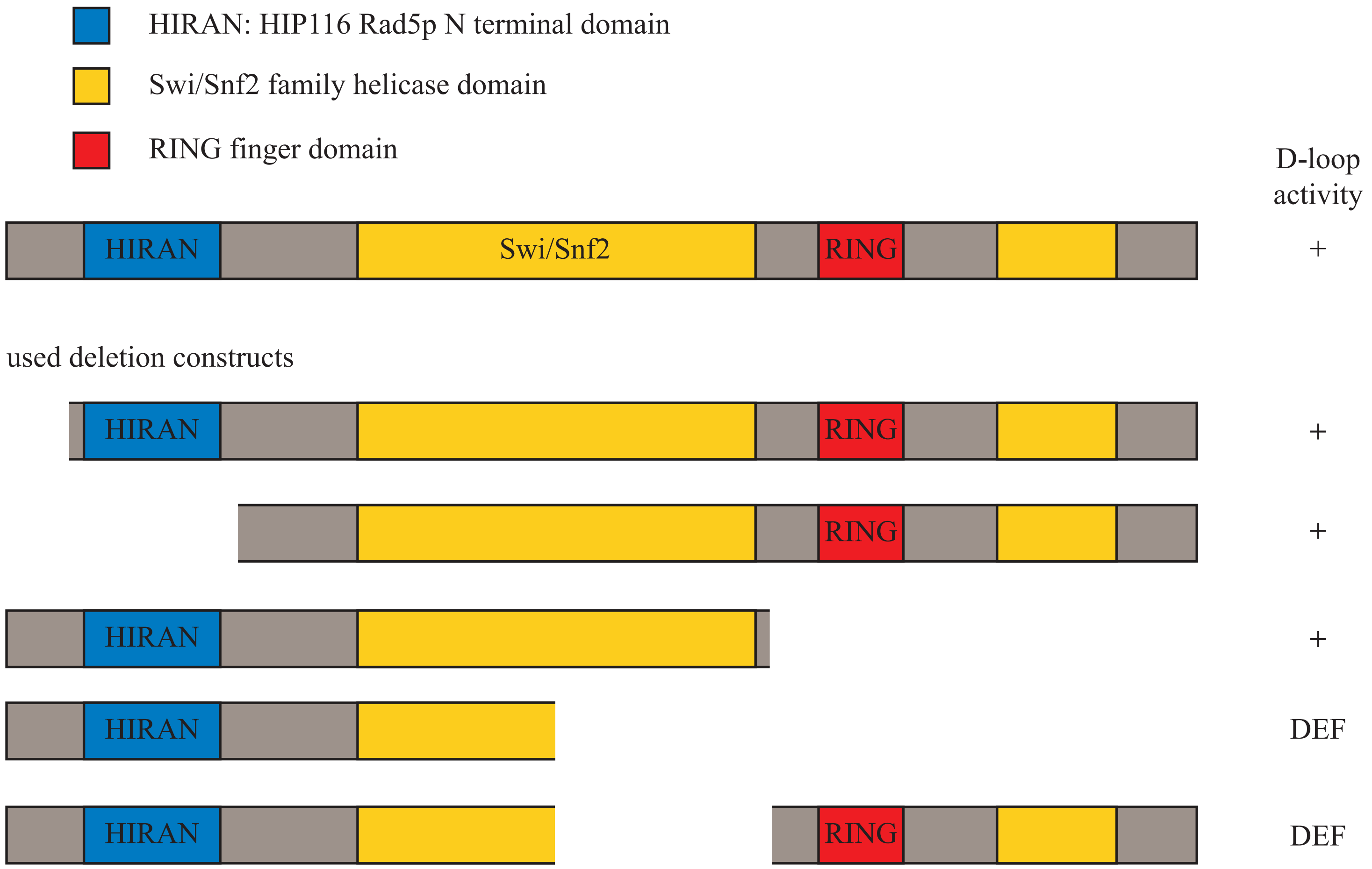

B

HLTF function	Swi/Snf2 domain	RING domain	HIRAN domain
PCNA polyubiquitilation	ND	+	ND
Fork reversal	+ (+ ATP)	ND	ND
double-stranded DNA translocase	+ (+ ATP)	ND	ND
protein remodelling	+ (+ATP)	ND	ND
D-loop forming	+ (- ATP)	-	-

Supplementary Figure 3

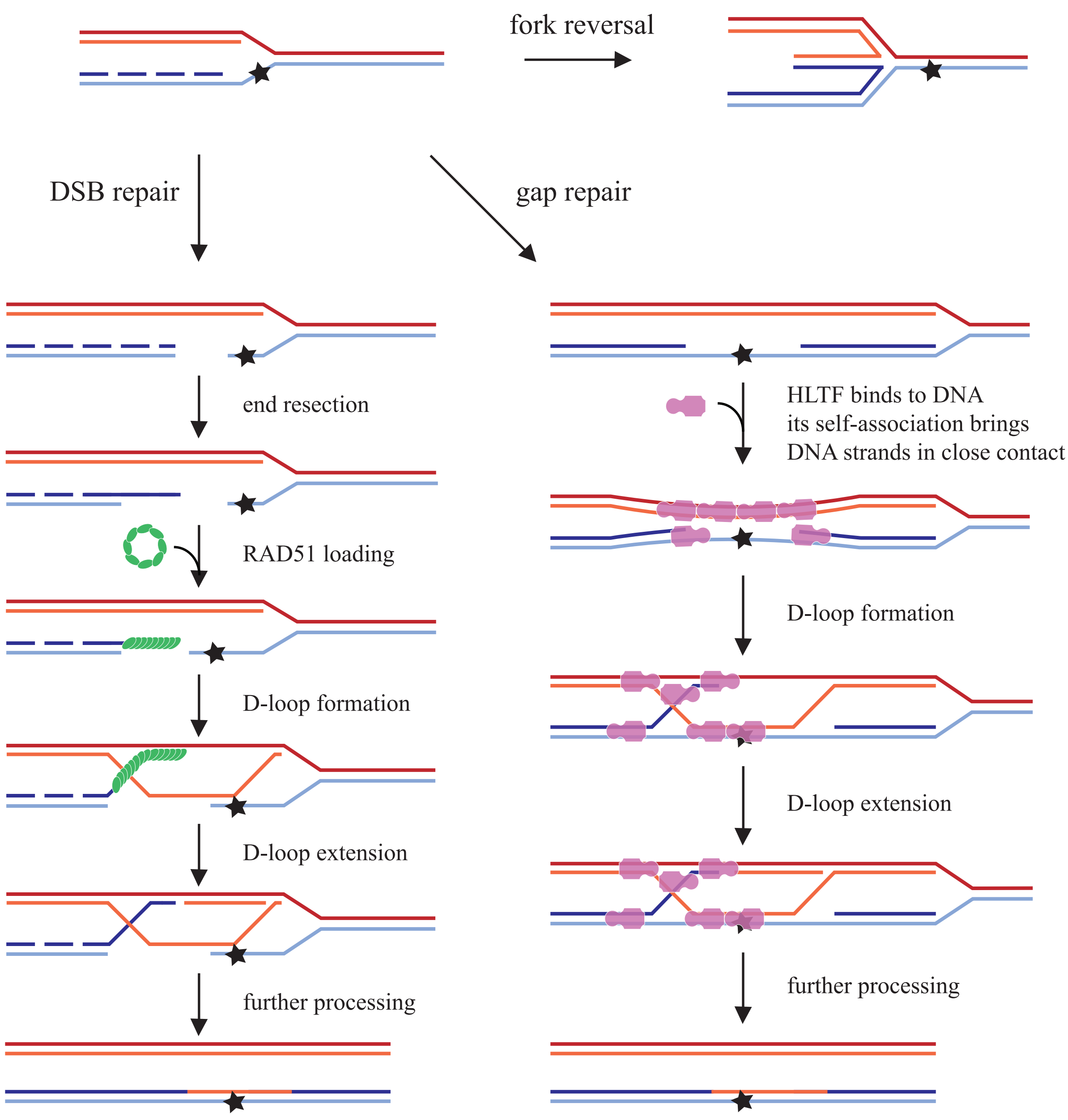

Supplementary Figure 4
